# Adaptive MHC-E restricted tissue-resident NK cells are associated with persistent low antigen load in alveolar macrophages after SARS-CoV-2 infection

**DOI:** 10.21203/rs.3.rs-1561222/v1

**Published:** 2022-04-25

**Authors:** Nicolas HUOT, Cyril Planchais, Vanessa Contreras, Beatrice Jacquelin, Caroline PETITDEMANGE, Marie Lazzerini, Pierre ROSENBAUM, Félix Rey, R. Keith Reeves, Roger Le Grand, Hugo Mouquet, Michaela Müller-Trutwin

**Affiliations:** institut pasteur; Institut Pasteur; CEA - Université Paris Saclay - INSERM U1184; institut pasteur; institut pasteur; institut pasteur; institut pasteur; Institut Pasteur; Beth Israel Deaconess Medical Center; Université Paris-Saclay, INSERM, CEA; Institut Pasteur; Institut Pasteur, HIV, Inflammation and Persistence Unit

## Abstract

Natural killer (NK) cells are innate lymphocytes with potent activity against a wide range of viruses. In SARS-CoV-2 infection, NK cell activity might be of particular importance within lung tissues. Here, we investigated whether NK cells with activity against Spike^+^ cells are induced during SARS-CoV-2 infection and have a role in modulating viral persistence beyond primary clearance from nasopharyngeal and tracheal tissues. We performed an integrated analysis of NK cells and macrophages in blood and bronchoalveolar lavage fluids (BALF) of COVID-19 convalescent non-human primates in comparison to uninfected control animals. SARS-CoV-2 protein expression was detected for at least 9–18 months post-infection in alveolar macrophages. Convalescent animals segregated into two groups based on cellular phenotypes and viral persistence profiles in BALF. The animals with lower persistent antigen displayed macrophages with a regulatory phenotype and enhanced MHC-E restricted NK cell activity toward cells presenting peptides derived from the SARS-CoV-2 Spike protein leader sequence, while NK cell activity from the other convalescent animals, control animals and healthy humans were strongly inhibited by these Spike peptides. The adaptive NK cell activity was not detected in blood but in tissue-resident NK cells, and cross-reacted against MERS-CoV and SARS-CoV Spike-derived peptides.

## Introduction

The alveolar membrane is the largest surface of the body in contact with the outside environment, making the lungs the most vulnerable organ of the whole body to microbial assault^1^. Protection of this barrier needs a rapid and effective immune response to prevent pathogen infections and spread. Concomitantly, the duration and the amplitude of the resulting inflammation must be effciently controlled to avoid tissue damage. From a global and historic perspective, the scope and scale of lower respiratory tract infections is greater than most other infections. Indeed, viral pneumonias are among the most lethal and pathologic human diseases. The current global outbreak of severe acute respiratory syndrome caused by the type 2 coronavirus (SARS-COV-2) is a dramatic contemporary example of the ability of viral pneumonias to rapidly disseminate and cause severe disease in human populations. SARS-CoV-2 replicates in both upper and lower airways^2^, and viral RNA is typically detected in patients 1–3 days before the onset of symptoms, with viral load in the upper respiratory tract peaking within the first week of infection, followed by a gradual decline over time. SARS-CoV-2 infection triggers cellular and humoral immune responses as well as activation of neural pathways, which contribute to distant inflammatory effects. Although it is generally considered that SARS-CoV-2 is an acute infection that does not become chronic, a growing number of studies suggest that some SARS-CoV-2-infected individuals do not clear the virus over long periods of time^3–6^.

Macrophages are innate immune cells that sense and respond to microbial threats by producing inflammatory molecules, phagocytosing pathogens and promoting tissue repair. A dysregulated macrophage response can be damaging to the host, as for instance in infection-induced macrophage activation syndrome as observed during SARS-CoV-2 infection^7,8^. Macrophages are abundant in the lung, comprising about 70% of the total leukocyte population^9^. Monocytes and macrophages can both be infected by SARS-CoV-2 through ACE2-dependent and ACE2-independent pathways^10–14^.

Natural killer (NK) cells are innate immune responders critical for viral clearance and immunomodulation. Interferon-alpha (IFN-α) is known for its activating role on NK cells. Despite their vital role in viral infections, the contribution of NK cells to SARS-CoV-2 immunity is not clear^15,16^. NK cell frequencies decrease in the blood during the early phase of SARS-CoV-2 infection^17–19^, and display an exhaustion phenotype^20–22^. NK cells from healthy individuals can directly kill SARS-CoV-2-infected cells *in vitro*, but NK cells from individuals with a moderate or severe disease displayed impaired NK cell activity^20^. Recent reports showed a correlation between high levels of NK cells in the blood and a rapid decline in viral load^20^. The rapid decrease of blood NK cells might be due to their death and/or to recruitment to the lungs or other tissues^23^. Previous studies on NK cells in other viral infections have clearly shown that tissue NK cells display distinct characteristics than NK cells in the blood. During SARS-CoV-2 infection, it is therefore essential to get insights on NK cells not only present in the blood but also in tissues, particularly in respiratory mucosal tissues.

The lung seems to provide an environment propitious for the development of adaptive-like NK cells^24^. Adaptive NK cells have been predominantly described during chronic infections, such as CMV and SIV infections^18,25–28^. A common denominator for the majority of adaptive NK cells is the expression of the activating heterodimeric receptor CD94/NKG2C that binds to HLA-E. The latter is a non-classical HLA molecule that displays a comparably restricted expression pattern, very limited polymorphism and binds hydrophobic nonamers^29^. The strength of the HLA-E/NKG2C interaction in conjunction with variable conditions of co-stimulation and a favorable cytokine milieu is considered to be the molecular basis for the complex inter- and intra-individual heterogeneity of adaptive NK cells. In CMV infection, presentation of the viral peptides *via* HLA-E on monocyte/macrophages might be key for a secondary expansion of NK cells^29^. An increase of adaptive NK cells in blood from patients with severe COVID-19 has been reported, although the identification of adaptive NK cells was based solely on cell phenotype without any functional analysis nor data in tissues^18^.

Here we took advantage of a non-human primate model (cynomolgus macaques) to evaluate NK cells in tissues after SARS-CoV-2 infection. We raised the hypothesis that adaptive NK cells arise preferentially in the lung in response to SARS-CoV-2 infection. We searched for SARS-CoV-2 derived peptides capable to bind to HLA-E and modulate NK cell activity. We investigated the presence of SARS-CoV-2-trained NK cells in the blood and lung. We performed functional assays to identify the presence of adaptive NK cells and evaluated whether they can be detected in the blood and/or in the lung. We analyzed whether the presence of Spike-specific adaptive NK cells is associated or not with viral persistence. Our study reveals the presence of viral antigen in alveolar macrophages up to at least 18 months after SARS-COV2 infection in macaques. Moreover, we uncover an association between lower level of persisting viral antigen in alveolar macrophages and the activity of Spike-specific adaptive NK cells in the lung.

## Results

### Persistent phenotypic alterations in bronchoalveolar macrophages from SARS-CoV-2 convalescent non-human primates

**Figure 1:**
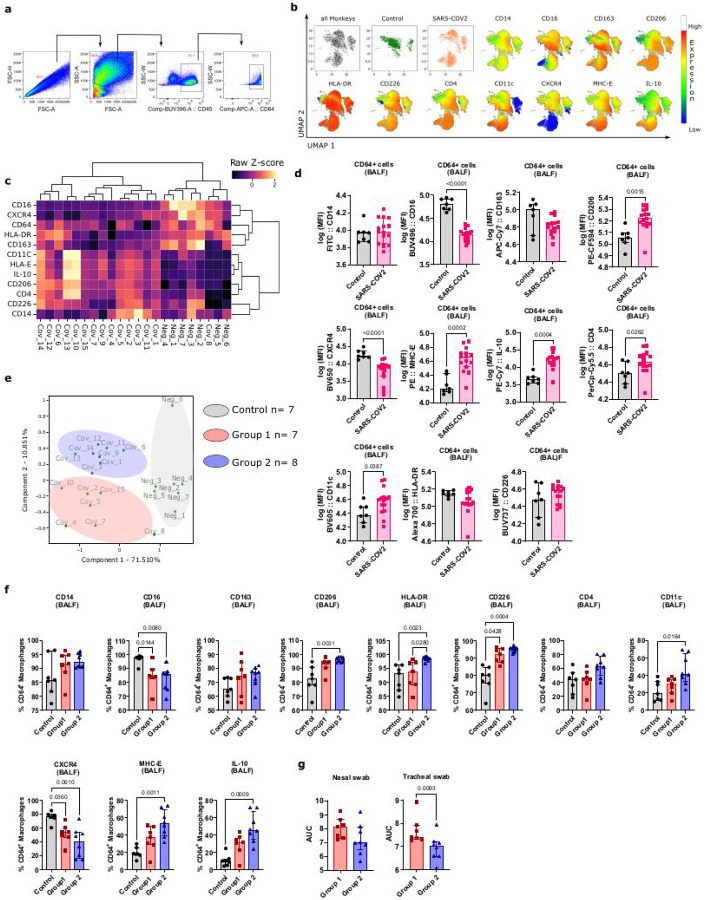
Immunophenotyping of bronchoalveolar macrophages in SARS-CoV-2 convalescent monkeys. **(a)** Representative flow cytometry plots showing the gating strategy for the bronchoalveolar macrophages. The latter were defined using FSC-A, FSC-H, SSC-A, and SSC-W parameters and surface expression of CD45 and CD64. (**b)** Singlet, live, CD45^+^CD64^+^ bronchoalveolar macrophages from seven and fifteen healthy and convalescent animals, respectively, were subjected to unsupervised analysis by the UMAP dimension reduction algorithm. Cells were gated, down sampled to 6,000 cells per sample which were barcoded and concatenated. CD45 and CD64 were excluded from the list of UMAP running parameters. The resulting UMAP projection is colored according to the status of the animals (green: healthy monkeys, orange: convalescent monkeys). UMAP plots showing expression intensities of each marker used in analysis are shown. (**c)** Hierarchical clustering heatmaps showing *z* score expression of indicated proteins in CD64^+^ BALF macrophages from control and convalescent monkeys. (**d)** Graph showing the Mean of Fluorescent Intensity (MFI) of the indicated markers expressed in CD64^+^ macrophages. Healthy monkeys (n=7) are in gray, convalescent monkeys (n=15) are in red. *p* values are determined by non-parametric Kruskal Wallis test **(e)** Principal component analysis (PCA) of CD64^+^ BALF macrophage-based expression levels of proteins shown in Fig.1**c**. Circles show clustering of different monkeys based on the PCA. Monkeys separated into three groups, the gray group contained all control monkeys (NEG_1 to 6), the pink colour corresponds to monkeys COV_10, 2, 15, 5, 7, 4, 8 and defines group 1, and the blue defines group 2 and contained monkeys COV_12, 11, 6, 14, 9, 1, 3, 13. **(f)** Graph showing the percentage of the indicated markers expressed in CD64^+^ macrophages. Healthy monkeys (n=7) are in gray, group 1 convalescent monkeys (n=7) are in pink and group 2 convalescent monkeys (n=8) are in blue. Monkeys were separated in two groups according to the groups defined in panel e; **(g)** Graph showing the air under the curve (AUC) based on the viral load measured during the acute phase in nasal and tracheal swab (see Supplemental Figure 1). Monkeys were separated in two groups according to the groups defined in panele. *p* values are determined by non-parametric Kruskal Wallis test. In each graph, each dot, square or triangle represents an individual monkey, bars represent the median, and the interquartile range is shown.

We aimed to investigate if viral antigens could persist over prolonged periods of time after SARS-CoV-2 infection in the lung and continue to stimulate immune cells. We used the nonhuman primate model to address this question in order to have access to tissues while still being as close as possible to the human infection and to human tissue parameters. Fifteen cynomolgus macaques were infected with SARS-CoV-2 (Wuhan strain) and followed between 6 and 18 months (Supplementary Table 1) as previously described^30^. The viral load (VL) in tracheal and nasal swabs reached median peak levels of 7.9×10^8^ copies/ml by day 3 post-infection (p.i.) (Suppl. Figure 1). All animals subsequently became PCR negative by day 14 p.i., and remained negative for the presence of SARS-CoV-2 RNA in nasal and tracheal swabs during the entire follow-up (Suppl. Figure 1). We investigated whether the virus could be present in other body parts of the animals after convalescence. Alveolar macrophages reside on the epithelial surface of the alveoli and are thus in direct contact with the environment and foreign particles entering the lungs, where they have been described to be infected by some pathogens, including bacteria and viruses^31–34^. To characterize the alveolar macrophages from the convalescent monkeys, we collected bronchoalveolar lavage fluids (BALF) from the 15 animals between 6 and 18 months after SARS-CoV-2 infection and compared them with BALF samples obtained from 7 healthy cynomolgus macaques that were never infected with SARS-CoV-2. We used multi-parameter flow cytometry and unsupervised analysis to investigate macrophage phenotypic pro les. The strategy for gating the macrophages is illustrated in Fig. 1a. Despite the long time after infection, we found that BALF macrophages segregated according to healthy and convalescent monkeys (all 15 animals except one) (Fig. 1b and 1c). Macrophages isolated from convalescent monkeys generally displayed higher levels of CD206, CD4, CD11c, CD226, MHC-E and IL-10 expression, and lower CD16 and CXCR4 expression when compared to healthy animals (Fig. 1b–d). Thus, macrophages from convalescent monkeys were clearly distinct from those in control monkeys.

We next compared the macrophage phenotypes between the convalescent animals. Using principal components analysis (PCA) based on 12 phenotypic parameters, convalescent monkeys also clustered separately from healthy controls. Moreover, convalescent monkeys segregated into two groups (Fig. 1e). Seven out 15 monkeys clustered together and were defined as group 1 (monkeys COV_2,_4,_5,_7,_8,_10 and _15), whereas the 8 other animals clustered in another group denominated here group2 (monkeys COV_1,_3,_6,_9,_11,_12,_13,_14) (Fig. 1e). Differences between the two groups of convalescent monkeys were driven by higher frequencies of macrophages expressing HLA-DR, CD11c, CD206, CD226, MHC-E and IL-10 in group 2 compared to group 1 and/or healthy monkeys (Fig. 1f and Supp. Figure 2a). Thus, in particular, group 2 macrophages showed a regulatory/M2 like phenotype.

Given the difference in the macrophage phenotype among convalescent monkeys, we analyzed whether there were differences in VL during primary infection between the two animal groups. We compared the area under the curve (AUC) of VL as quantified by PCR in the nasal and tracheal swabs during the acute phase of the infection between each group (Supplementary Fig. 3). The AUC VL were higher in group 1 than group 2 in tracheal swabs (Fig. 1g). Group 1 also showed a trend for higher VL in tracheal swabs at day 1 p.i as compared to group 2 (p = 0.09). Collectively, our results show that BALF macrophages displayed profound alterations even after a long period after SARS-CoV-2 infection, suggesting an imprinting of the alveolar macrophages. Of note, monkeys with the strongest imprinted pro le toward regulatory M2-like macrophages (group 2) were those showing the lowest VL during acute infection.

#### Detection of SARS-CoV-2 protein in alveolar macrophages one year post-SARS-CoV-2 infection

**Figure 2:**
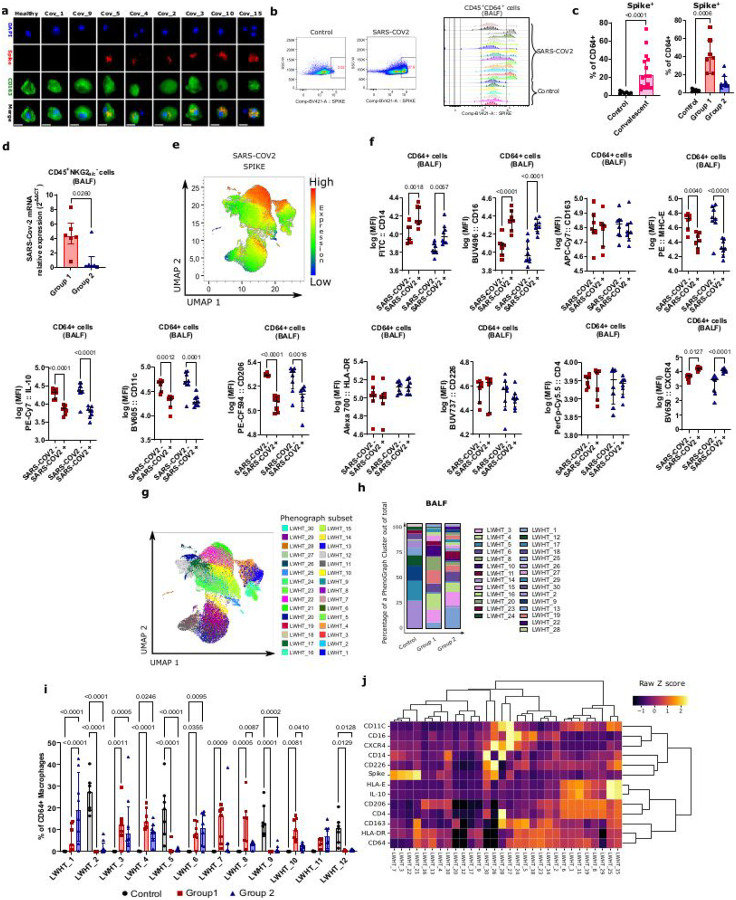
Bronchoalveolar macrophages harbor Spike proteins in SARS-CoV-2 convalescent monkeys. **(a)** Fluorescence microscopy on fixed primary BALF macrophages after 24h of *in vitro* culture. CD45^+^CD64^+^ cells isolated from BALF of all convalescent monkeys were stained for nuclei (DAPI, blue), CD163 (anti-CD163-Alexa Fluor 488; green) and Spike protein (anti-Spike protein, Texas red). Representative stainings from eight animals are presented in the Figure. Scale bar: 20 μm. (**b)** (Left) Flow cytometry plots showing SARS-COV-2 intracellular staining in BALF macrophages for one representative healthy and one representative convalescent monkey. (Right) Histograms of intra-cellular Spike SARS-CoV-2 expression on CD64^+^ BALF macrophages. Each animal is represented on the histogram. (**c)** Percentage of CD64^+^ macrophages positive for SARS-CoV-2 Spike proteins. (**d)** Relative levels of SARS-CoV-2 RNA in BALF cells (**e)** UMAP plots showing expression intensities of intracellular SARS-CoV-2 Spike expression in CD64^+^ macrophages of BALF. (**f)** Comparison of the MFI of the indicated markers between Spike+ and Spike- macrophages for a given group. A Šídák’s multiple comparisons test was applied. **(g)** PhenoGraph clusters overlaid on the UMAP plot. (**h)** Frequence of the 30 PhenoGraph clusters within CD64^+^ macrophages. (**i)** Distribution of the most frequent PhenoGraph clusters for each group. (**j)** Hierarchical clustering of PhenoGraph clusters. The heatmap was calculated as Raw *z* score of the indicated markers within the clusters. *p* values are determined by non-parametric Kruskal-Wallis test in graph **c** and **d**. A Tukey multiple comparisons test was used to determine the P value in graphs **i**.) Interaction F (DFn, DFd) is F (58, 570) = 9,977, Row Factor F is (29, 570) = 8,462 and Column Factor F is (2, 570) = 1,384e-005. In each graph, each dot, square or triangle represents an idividual monkey, bars represent the median, and the interquartile range is shown. Control monkeys are in gray, monkeys from the group 1 and 2 are in pink and blue respectively.

We then investigated whether SARS-CoV-2 could be present in the lung despite the fact that the animals became PCR negative in nasal and tracheal swabs by probing viral antigens in alveolar macrophages of the 15 convalescent monkeys. To this end, we performed an antibody-based fluorescent staining of SARS-CoV-2 Spike in alveolar macrophages *in vitro*, and detected Spike protein expression to variable extents in the animals (Fig. 2a). To quantify the frequency of macrophages harboring viral Spike protein and its expression level, we performed *ex-vivo* intracellular flow cytometry staining of Spike protein in BALF cells from the 15 convalescent and 7 control monkeys (Fig. 2b). The median frequency of Spike^+^ macrophages was 21.3%, ranging from 6.1 to 72.7% depending on the animal (Fig. 2c). The seven monkeys from group 1 showed the highest frequencies of Spike^+^ macrophages (median = 38.80%), and were among the 9 animals displaying frequencies around or above the median levels (Fig. 2c). To confirm the presence of the virus, we searched for viral RNA in the BALF cells. A RT-qPCR was applied to all available samples (6 animals per group). SARS-CoV-2 RNA was detected in BALF cells to a variable extent among the animals (Fig. 2d). Viral RNA levels were higher in group 1 than group 2 animals (Fig. 2d). Thus, these analyses allowed identifying animals with high frequencies of alveolar macrophages positive for SARS-CoV-2 Spike protein and viral RNA even after 6–18 months post-infection, in particular in group 1.

### Spike^+^ alveolar macrophages display a distinct phenotype

We next aimed at determining the phenotype of BALF macrophages harboring Spike proteins. SARS-CoV-2^+^ macrophages were gated manually and projected onto a Uniform Manifold Approximation and Projection (UMAP) plot as shown in Fig. 1b. Most Spike^+^ macrophages clustered together (Fig. 2e). Spike^+^ macrophages from both groups expressed higher levels of CD14, CD16, CXCR4, and lower levels of CD206, CD11c, MHC-E and IL-10 as compared to Spike^–^ macrophages (Fig. 2f).

To identify subsets of Spike^+^ macrophages and to quantify potential inter-group differences in their relative abundance, a PhenoGraph algorithm was applied to the BALF macrophages rendering 30 distinct clusters (Fig. 2g and h). Among them, clusters LWHT_2, 5, 9, 12, 14,18, 23 and 24 were predominantly found in the control monkeys (Fig. 2h–i and Supplementary Fig. 4a). Their BALF macrophages were characterized by a stronger expression of CD16 and CD64, CD163 and HLA-DR (Fig. 2j). Group 1 contained higher frequencies of clusters LWHT_3, 4, 6, 7, 8 and 10 compared to control animals (Fig. 2h–i and Supplementary Fig. 4a). The clusters LWHT_3 and 7 expressed high levels of the Spike protein and corresponded to macrophages expressing higher levels of CD14 and low levels of CD4, CD206, IL-10 and MHC-E (Fig. 2j). Consistent with these findings, the tracheal swab VL (AUC) in acute infection correlated positively with CD14 and CXCR4 expression (p = 0.05, r = 0.52 and p = 0.025, r = 0.58, respectively) (Supplementary Fig. 4b). The other clusters with high level of Spike proteins were LWHT_21, 22, and 26, but their frequencies were below 0.43, 0.1 and 0.27% respectively (Supplementary Fig. 4a). Group 2 displayed higher levels of clusters LWHT_1 compared to Group 1 and control animals. Cluster LWHT_1 corresponded to macrophages expressing high levels of CD4, CD206, IL-10 and MHC-E (Fig. 2i). When comparing the expression levels of all markers to each other, IL-10 and MHC-E expression were strongly correlated (p = 1.89e-^7^, r = 0.95), further indicating that they are expressed by the same subpopulations (Supplementary Fig. 4b). Altogether, most macrophages harboring Spike protein displayed a similar phenotype, corresponding to the predominant macrophage phenotype in group 1 and distinct from the regulatory-like macrophages in group 2.

### Elevated NK cell levels in BALF of group 2 convalescent monkeys

We next assessed whether NK cells differed between group 1 and group 2 convalescent monkeys. NK cells were gated as commonly described for macaque NK cells (i.e. CD45^+^CD3^–^CD20^–^NKG2_A/C_^+^)^35,36^ (Supplementary Fig. 5a). In Old world monkeys, such as macaques, most NK cells express NKG2^37^, but the antibodies available for these species do not differentiate between NKG2A and NKG2C and we therefore used the nomenclature NKG2A/C here. We first analyzed if NK cell tissue distribution was altered 6–18 months post-infection. NK cells were quantified in the lungs (BALF), blood and bone marrow by flow cytometry (Supplementary Fig. 5a). NK cell frequencies in blood from group 1 convalescent monkeys were higher than in healthy and group 2 monkeys (Fig. 3a). Conversely, NK cell frequency in BALF was higher in group 2 than in healthy and group 1 monkeys (Fig. 3a). No difference was seen regarding bone marrow NK cell frequency between the groups (Fig. 3a).

**Figure 4:**
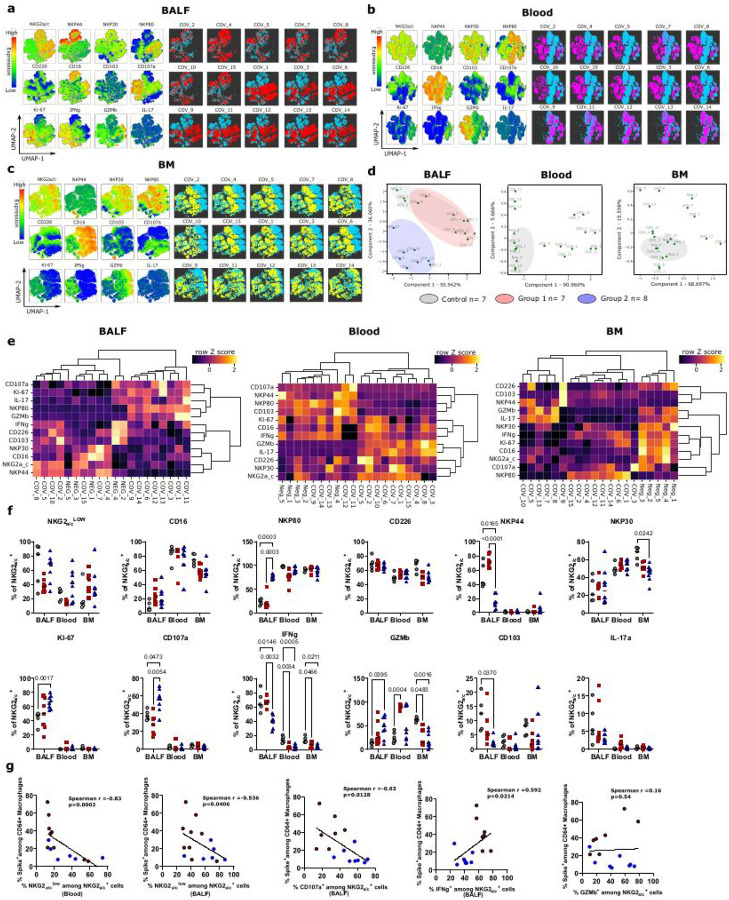
Immunophenotyping of tissue NK cells in SARS-COV 2 convalescent monkeys. **(a-c)** UMAP showing NKG2_a/c_^+^ cells of control (n=5) and convalescent monkeys (n=15) isolated from BALF (**a**), blood (**b**) and bone marrow (BM) (**c**). NKG2_a/c_^+^ tissue NK cells were subjected to unsupervised analysis by the UMAP dimension reduction algorithm. Cells were gated, down sampled to 6.000 cells per sample, which were barcoded and concatenated. For each panel, the right part shows UMAP plots showing expression intensities of each marker, and the left part represent mapping of NK cells from each monkey on the UMAP plot. In each UMAP plot, blue dots represent concatenated healthy monkeys for each tissue, and pink dots represent lung NK cells (**a**), purple dots represent blood NK cells (**b**), and yellow dots represent BM NK cells (**c**), from the indicated convalescent monkeys. NKG2_a/c_ was excluded from the list of UMAP running parameters. (**d)** PCA of BALF (**left**), blood (**middle**) and BM (**right**) based on protein expression levels shown in Figure (**a-c**). Each dot represents a monkey, full circles show clustering of monkeys based on the PCA performed on NKG2a/c^+^ NK cells for the indicated tissue. **(e)** Hierarchical clustering heatmaps showing row *z* score expression of indicated proteins from NK cells of convalescent monkeys compared to healthy controls in BALF (**left**), blood (**middle**) and BM (**right**). (**f)** Graph showing the percentage of cells expressing the indicated markers among NK cells in the given tissues. Tukey multiple comparisons test was used to determine the P value. (**g)** Spearman’s correlations between CD64^+^ Spike^+^ macrophages and the frequency of the indicated NK cell populations. In each graph, each dot, square or triangle represents an individual monkey, bars represent the median, and the interquartile range is shown. Control monkeys are in gray (n=5), animals from the group 1 in pink (n=7), and animals from group 2 in blue (n=8). BM= bone marrow.

Since we observed different NK cell frequencies in the blood and BALF depending on the animal group, we next determined by flow cytometry the frequency of major traffcking receptors for NK cells (CCR7, CD62L, CXCR3 and CXCR4) in the blood and BALF. CXCR3 is considered as the major homing receptor for human NK cells to the lung, also during SARS-CoV-2 infection^38,39^. The frequencies of CXCR3^+^ and CCR7^+^ NK cells in BALF were higher in group 2 than group 1. There was no difference regarding CD62L between the groups (Fig. 3b and 3c). This pattern is indicative of a higher capacity of NK cells from group 2 to home to the lung compared to group 1. However, CXCR3^+^ NK-cell frequencies were increased in blood of group 1 as compared to group 2 and control animals. Additional mechanisms could participate to the differences between animals in term of NK cell distribution pattern. We reasoned that the increases of NK-cell frequency in lung of convalescent monkeys from group 2 might also be due to *in-situ* expansion of resident NK cells. Therefore, we next analyzed markers linked to tissue residency. By analogy with tissue resident human T lymphocytes and NK cells, we investigated the expression of CD69, the integrin CD49a and CD103 (Fig. 3d). NK cells in BALF expressing the tissue residency markers CD49a or CD69 were increased in all convalescent monkeys or in group 2, respectively. We also analyzed Ki-67 expression as a marker of recent proliferation. The NK cells from BALF showed higher expression levels of Ki-67 than those from the blood or bone marrow (Fig. 3d and Fig. 4a–b). Frequencies of Ki-67 NK cells were higher in group 2 than group 1 in BALF. Together, these results demonstrate altered tissue distributions of NK cells in the convalescent monkeys. They indicate that NK-cell homing behavior differs between convalescent and healthy monkeys. NK cells from group 1 animals were increased in blood but not in BALF suggesting an impaired homing capacity to the lung. In contrast, NK-cell frequencies were increased in BALF of group 2 as compared to the other animals, and this was associated with a higher tissue homing receptor expression (CXCR3, CCR7), stronger proliferation (Ki-67) and lung tissue residency markers (CD49a, CD69) suggesting *in-situ* differentiation.

### Convalescent animals with regulatory-like macrophages also harbor distinct NK cells in BALF

To determine if SARS-CoV-2 infection can have a long-term impact on the function and composition of the NK-cell pool, flow cytometric analysis of NK cells from blood, BALF and bone marrow was performed including 12 phenotypic markers of function and tissue residency *via* an unsupervised approach. We applied an UMAP analysis on tissue NK cells (Fig. 4a–c). PCA analysis showed that monkeys belonging to group 2 clustered together and harbored phenotypic NK-cell populations clearly distinct from control and group 1 animals in BALF (Fig. 4d). Conversely, monkeys in group 1 displayed NK-cell populations in BALF similar to those in control monkeys (Fig. 4d). The same approach performed on NK cells from blood and bone marrow showed differences between control and convalescent animals but did not allow to cluster the animals into groups (Fig. 4d). Thus, NK cells in BALF clustered distinctly according to the tissues. In BALF, NK cells from group 2 were more distinct from control animals than group 1.

We further dissected the segregation pro les of NK-cell populations (Fig. 4e and Supplemental Fig. 5a and 5b). In BALF, group 2 monkeys showed decreased frequencies of NK cells expressing NKp44 and IFN-g, and increased levels of NKP80 + and CD107a^+^ NK cells when compared to control and group 1 monkeys, as well as increased levels of cells expressing Ki-67 and GZMb when compared to control monkeys (Fig. 4f). Thus, NK cells from group 2 segregated from the other animals in BALF and displayed a highly differentiated, cytotoxic phenotype.

We next asked whether there is a relation between the frequency of Spike^+^ macrophages in BALF and the NK cell phenotype. We analyzed the potential correlations between the levels of Spike^+^ macrophages and NK cells expressing functional markers (IFN-g, CD107a, GzB) and/or markers of education (NKG2A/C) (Fig. 4g). Both in the BALF and blood, NKG2A/C^low^ NK cells, which are generally considered as cells with a more educated phenotype^40,41^, correlated negatively with the levels of Spike^+^ macrophages. In addition, CD107a NK cells in BALF also correlated negatively with Spike^+^ macrophage frequencies (Fig. 4g). In contrast, IFN-g^+^ NK cells correlated positively with the frequency of Spike^+^ macrophages in BALF (Fig. 4g). Thus, NK cells with a phenotype frequently found in group 1 correlated positively with macrophage-associated persisting antigen load, while the cytotoxic NK cells from group 2 correlated negatively with the antigen load.

#### Peptides derived from the Spike protein leader sequence bind to HLA-E and inhibit NK cell activity

**Figure 5:**
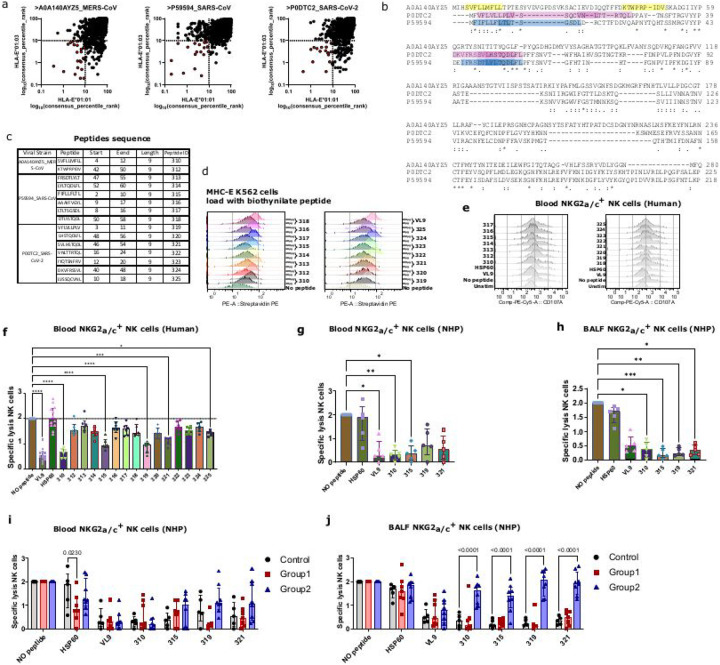
HLA-E dependent cytotoxic activity of NK cells. **(a)** HLA-E binding predictions using the IEDB analysis resource. This is a T cell epitope prediction tool, which takes into account an amino acid sequence and determines each subsequence’s ability to bind to a specific MHC class I molecule. Here the sequence corresponds to entire spike protein. The multiple possible subsequences of it are represented by the black dots in the graph. Each peptide and its predicted capacity for binding to HLA-E is provided in the Source Data le. The location of each dot represents the predicted capacity of binding to HLA-E. For each peptide, percentile ranks for binding to HLA-E*0101 are indicated on the y-axis, and percentile ranks for binding to HLA-E*0103 are shown on the x-axis. A low percentile rank value indicates high affinity. The selected Coronavirus peptides are shown in red. **(b)** Amino acid sequence alignment of the Spike protein from MERS-CoV (UniProtKB-A0A140AYZ5), SARS-COV-1 (UniProtKB-P59594), and SARS-CoV-2 (UniProtKB-P0DTC2). Regions corresponding to selected 9-mers are indicated by yellow, purple, and blue boxes. Asterix (*) indicates positions with a single, fully conserved residue. Colon (:) positions with conservation between amino acid groups of similar properties and period (.) positions with conservation between amino acid groups of weakly similar properties. **(c)** Table showing the selected nonameric sequences of peptides in the Spike protein of the 3 analyzed Coronavirus viruses showing similarities with the canonical MHC-E binding motif, such as strong hydrophobicity and a Leucine in the last position. (**d)** Each histogram shows HLA-E expression after loading K562-E*0101 cells with the given peptide. Each curve indicates the mean fluorescent intensity (MFI) of HLA-E on K562-E*0101 cells loaded with the indicated peptide for a given concentration. VL9 is a control peptide well known to bind to MHC-E and deriving from classical HLA-I. One representative experiment out of three is shown. (**e)** Histogram showing CD107a surface expression on human NKG2_a/c_^+^ NK cells after 8 h of co-culture with K562-E*0101 cells loaded with the indicated peptide. Data from one representative donor are shown. (**f)** HLA-E dependent cytotoxic activity of human NKG2a^+^ NK cells isolated from blood of healthy donors against distinct Coronavirus peptides. Each dot, square or triangle represents an individual monkey. (**g-h)** HLA-E dependent cytotoxic activity of NKG2_a/c_^+^ NK cells isolated from blood (**g**) and BALF **(h)** of healthy monkeys against distinct coronavirus peptides. Each symbol in the figures represents an individual monkey. (**i-j**) HLA-E dependent cytotoxic activity of NKG2_a/c_^+^ NK cells isolated from blood (**i**) and BALF **(i)** of healthy monkeys (grey) and Group 1 and 2 of convalescent monkeys (pink and blue respectively) against distinct Coronavirus peptides. In each graph, asterisks indicate significant p value. Bars indicate median values, and error bars indicate the interquartile range. Cytotoxic activity of NK cells in various conditions was compared to the condition without peptide using the Wilcoxon test for paired data.

Next, we aimed to analyse whether NK cells of group 2 harbor a stronger antiviral activity. Based on our results showing an up-regulation of MHC-E on macrophages in group 2, we first investigated whether NK cells in group 2 were trained to specifically recognize Spike^+^ target cells in a MHC-E dependent manner. We set up a functional assay to analyze MHC-E restricted viral suppressive activity of NK cells toward target cells presenting Spike antigens *via* MHC-E. To this end, we searched for SARS-CoV-2-derived peptides with a high probability of binding to MHC-E. We analyzed the whole Spike protein but focusing on the leader sequence (LS), assuming a higher probability in identifying peptides binding to MHC-E *in vivo* in analogy to the cellular peptides that generally bind to MHC-E and that derive from the LS of classical MHC class I molecules^42^ (such as the VL9 peptide) or from the LS of the stress protein Hsp60^43^. We analyzed the Spike LS of SARS-CoV-2 as well as those of MERS-CoV and SARS-CoV-1. We used the Immune Epitope Data Base (IEDB) tool to predict the capacity of Spike-derived peptides to bind to HLA-E*0101 and HLA-E*0103, the only two functional HLA-E alleles in humans. We found > 60 nonamer peptides (out of 1169, 1087 and 1097 analyzed Spike peptides from MERS-CoV, SARS-CoV-1 and SARS-CoV-2, respectively) showing a probability to be loaded by HLA-E (Fig. 5a). Among those, we selected peptides encoded by sequences similar to the canonical sequence motif of HLA-E binding peptides^44^. In this way, 2, 6 and 7 peptides were identified for MERS-CoV, SARS-COV-1 and SARS-CoV-2, respectively (Fig. 5b and 5c). These peptides were all localized in similar regions of the LS from all three viruses (between amino acid positions 2–11, 10–24 or 40–56) (Fig. 5b). The 15 Spike LS-derived peptides were then analyzed for their capacity to stabilize HLA-E at the cell surface (HLA-E needing to bind peptides to be stably presented at the cell surface). The viral peptides increased in a concentration dependent manner the HLA-E expression on MHC-I devoid K562 cells stably transduced with HLA-E*0101 (K562-E*0101) (Fig. 5d), indicating that these peptides are able to bind HLA-E. HLA-E expression levels varied depending on the peptide, suggesting distinct HLA-E biding properties between the peptides.

We then analyzed NK cell activity against target cells presenting Spike LS-derived peptides *via* HLA-E. To validate the assay, we first studied NK cells from human healthy donors. These donors were all naïve of SARS-CoV-2 infection or vaccination. NKG2A^+^ NK cells from the donors were co-cultured with K562-E*0101 cells pre-loaded or not with the peptides, and NK-cell degranulation activity was measured by measuring CD107a expression (Fig. 5e and 5f). As expected, the HLA-I-derived VL9 control peptide inhibited NK cell activity, while the control Hsp60 peptide did not, validating our assay (Fig. 5f). When pulsing cells with the viral peptides, NK cell activity was modulated in a peptide dependent manner. For instance, peptide 310 from MERS-CoV, peptide 315 from SARS-CoV-1 and peptides 319, 321 and 325 from SARS-CoV-2, blocked NK-cell degranulation, whereas the other peptides did not. Of note, peptides 310, 315 and 319 all mapped in the N-terminal portion of the LS for each virus (positions 2–11) (Fig. 5b), suggesting a key role of this region in inhibiting the MHC-E dependent NK-cell activity.

We next performed the assay using NK cells from the control monkeys, and focused on the peptides having shown the capacity to modulate human NK-cell activity (peptides 310, 315, 319 and 321). As expected, the VL9 peptide inhibited the NK cells isolated from the blood of control monkeys, while the HSP60 peptide did not (Fig. 5g). Two out of the four peptides (peptides 310 and 315) blocked degranulation similarly to VL9 (Fig. 5g). NK cells isolated from BALF of control monkeys were inhibited by SARS-CoV-2 peptides even more strongly than those from the blood as the four viral peptides inhibited their degranulation (Fig. 5h).

### NK cells with enhanced MHC-E restricted Spike-specific activity in BALF of group 2 convalescent animals

Since we observed changes in the NK-cell phenotype in the convalescent monkeys as compared to healthy monkeys, and in particular signs of more educated and cytotoxic NK cells in group 2, we compared the MHC-E dependent suppressive activity of NK cells isolated from convalescent monkeys. The MHC-E dependent suppressive activity of NK cells in blood was similar between control and convalescent monkeys (Fig. 5i). Thus, the LS-Spike peptides also inhibited activity of NK cells from convalescent monkeys in blood. We then analyzed NK cells from BALF. MHC-E-dependent NK-cell activity was inhibited by the LS-spike peptides in healthy and group 1 convalescent monkeys (Fig. 5j). In contrast, NK cells isolated from group 2 showed a strong activity against all LS-Spike peptides tested (Fig. 5j). Altogether, we identified peptides derived from the LS of the SARS-CoV-2 Spike protein inhibiting NK cells from healthy monkeys and humans. In particular, the N-terminal part of the Spike LS encoded peptides were inhibitory for MHC-E dependent NK cell activity. Moreover, our data unraveled that SARS-CoV-2 infection can induce NK cells with enhanced MHC-E restricted activity against cells presenting Spike-antigens. This adaptive NK-cell activity was enhanced for half of the animals (group 2). It was observed in BALF but not in the blood. Moreover, it was cross-reactive against the LS-Spike peptides from other Coronaviruses.

## Discussion

Innate immune responses have been shown to play a vital role against COVID-19, including IFN-a and TGF-b whose role consists, among others, to modulate NK cell responses^20,45,46^. However, more information is needed on the role of cellular innate immunity, particularly in tissues, during SARS-CoV-2 infection. The tissue-specificity of macrophages and NK cells, as well as their plasticity and function have been investigated here in tissues of a non-human primate model of SARS-CoV2 infection. We found profound changes in the NK cell repertoire and macrophage phenotype in convalescent monkeys that indicated imprinting of macrophages and training of NK cells. The convalescent animals separated into two groups based on macrophage phenotype and viral protein levels. SARS-CoV-2 protein was detected up to 18 months post-infection in alveolar macrophages to variable levels. The animals with lower viral proteins displayed strong MHC-E restricted NK cell activity toward cells presenting peptides derived from the LS of the SARS-CoV-2 Spike protein, while NK cell activity from the other convalescent animals and from healthy humans and control animals were strongly inhibited by these Spike LS-derived peptides. The adaptive NK cell activity was not detectable in blood but detected in tissue-resident NK cells. It was specific for the Spike peptides, and remarkably, was cross-reactive against MERS-CoV and SARS-CoV spike-derived peptides.

One major finding in this study is the frequent detection of viral antigens in lung macrophages after a long period post-infection. Thus, viral antigen might persist more frequently than initially expected. Our data indicating persistence of SARS-CoV-2 virus in BALF macrophages are consistent with reports showing that SARS-CoV, MERS-CoV and SARS-CoV-2 can infect human macrophages *in vitro*^10–13, 47^. The capacity of macrophages to express ACE-2 in vivo has been previously shown from prehypertensive patients^48^, and recent reports on post-mortem findings in patients succumbing to COVID-19 showed ACE2 expression and viral nucleocapsid protein in CD169^+^ macrophages from lymph nodes, spleen and lung^49–52^, arguing in favor of the capacity of SARS-CoV-2 to directly infect macrophages *in vivo*. Presence of viral antigen and viral RNA in macrophage infection might also result independently of ACE2 expression from phagocytosis of infected alveolar epithelial cells, followed or not by escape from the lysosome. Antibody-dependent enhancement of cellular infection has been suggested as an additional mechanism of macrophage infection based on data in cellular and animal models of SARS-CoV and MERS-CoV infection^53,54^. Regardless of the source of the Spike-protein and the mechanism of entry into macrophages, our study clearly demonstrates the presence of SARS-CoV-2 antigens in the lung of monkeys up to 18 months post-infection.

Here, the Spike^+^ macrophages in BALF were those that expressed higher levels of CD14 and CD16, suggesting that they could have derived from infiltrating inflammatory monocytes. Recent studies reported a significant number of non-classical monocytes containing SARS-CoV-2 S1 protein in both severe and post-acute sequelae of COVID-19 (PASC) patients up to 15 months post-infection^55^. It is also possible that macrophages became infected in acute infection and then survived for many months. The viral antigen was detected in alveolar macrophages despite the fact that the virus was not detectable in the nasopharyngeal and tracheal swabs. This correlates with other studies in human post-mortem tissues and animal models reporting detection of persisting SARS-CoV2 in other parts of the body, such as lung, the central nervous system and olfactory bulbs^56–58^. Our results are in agreement with previous models proposing that SARS-CoV-2 initially infects and replicates in epithelial cells in the nasopharynx, which express relatively high levels of *ACE2* compared with epithelial cells in lower airways or the distal lung^59,60^. Whether by progressive movement distally in the tracheobronchial tree or via aspiration of nasopharyngeal contents, some virus gains access to the distal alveolar space. In the alveolar space, SARS-CoV-2 infects alveolar epithelial cells and tissue-resident alveolar macrophages^61^. Thus, our results support the increasing data in the literature indicating that the virus or viral antigens can persist for long time after infection in lung tissues. This has important implications, because the viral antigens can thus be recognized by and stimulate other cells of the immune system in the lung during a long period.

Many viruses have been described to persist and/or to establish a latent infection in macrophages. Members of Togaviridae, the Chikungunya alphavirus (CHIKV) and the Ross River virus, have been shown to persist in macrophages for a long time. The CHIKV can persist in synovial tissue in humans 18 months after infection^62^, with the productive replication of virus in synovial macrophages^63^. The Ross River virus showed high level of replication in macrophages shortly after infection, but viral antigens were also detectable by IFA analysis after 170 days in an *in vitro* model, indicating that the virus had not been completely cleared from the cells^64^. Long persistence in liver macrophages was observed for a member of the Flaviviridae family—the hepatitis C virus, albeit in small quantities, up to 9 years after therapy^65^. Ebola virus has been reported to persist in sperm and eye^66^. Productive virus release of HCMV from monocyte/macrophages is detectable up to 16 weeks after infection^67^. Similar to other viruses, SARS-CoV-2 virus might thus exploit the cell biology of monocyte/macrophages as a repository for dissemination and long-term persistence.

The present study demonstrates that the phenotype of the alveolar macrophages in the convalescent animals 6–18 months post infection was different from the non-infected animals, suggesting that SARS-CoV-2 infection has imprinted the macrophages. The alveolar macrophages from the convalescent animals displayed a M2/regulatory-like phenotype, expressing for instance IL-10. Alveolar macrophages are for the most part generally derived from fetal monocytes that colonize the lung shortly after birth, but are replenished by blood-borne monocytes if they are damaged or depleted^68^. During SARS-CoV-2 infection, tissue damage in lung is often present. Regulatory macrophages have been reported to be involved in the tissue repair^69,70^. The induction of such cells might thus be part of a normal process of healing. It is interesting to note that the phenotype of regulatory macrophages was most pronounced in the group of animals with lower levels of initial viral load and lower levels of persistent viral antigen.

The regulatory-like macrophages expressed higher levels of MHC-E. We reasoned that they either contributed to educate NK cells or that animals with a stronger MHC-E restricted viral suppressive capacity of NK cells after SARS-CoV-2 infection were more efficient in eliminating cells that present viral antigens via MHC-E. Our study reveals indeed an enhancement of MHC-E restricted viral suppressive NK cell activity in the group of animals with the lower viral load. This activity was directed tOfficells presenting peptides derived from the LS of the Spike protein. It has previously been shown that Spike-derived peptides can bind to MHC-E^71^. However, we identified and analyzed here peptides specifically derived from the LS in the spike protein and showed that the ones from the N-terminal region were particularly strong in inhibiting the NK cell activity of animals and human donors who had never previously seen SARS-CoV-2 antigens. Strikingly, after SARS-CoV-2 infection, the NK cells from about half of the animals were capable to adapt and were not any more inhibited by the Spike LS peptides. It is unclear, if the enhanced MHC-E restricted NK cell activity was the consequence or the cause of the lower viral load in early infection and/or after acute infection. Future studies will need to determine why some animals showed the enhanced MHC-E restricted NK cell activity, while others didn’t. It might be related to early events in the infection, such as a particular cytokine environment induced upon infection. The cytokines produced during Sars-CoV-2 infection may indeed influence the generation of adaptive NK cells and explain the differences observed between convalescent monkeys, similar to what has been observed previously in antigen-dependent^72,73^, antigen-independent, and cytokine-dependent NK cell recall responses in humans^72,74–76^.

The enhanced NK cell activity against LS-spike was only observed in group 2 animals, that also showed higher levels of regulatory-like macrophages, expressing high levels of MHC-E. An *in vivo* priming of the NK cells through antigens presented by macrophages is not excluded and has been suggested for chronic viral infections^79^. The crosstalk with monocytes or macrophages and the nature of ligands/receptors implicated are just emerging, and the cross-talk between M2 and NK cells remains ill-defined^77–80^. Here, NK cells in BALF from group 2 were highly differentiated and frequently expressed the maturation marker NKP80, that is involved in NK/macrophage crosstalk^81–83^. Thus, it is possible, that the microenvironment and/or cross-talk between macrophages and NK cells favored the emergence of adaptive NK cells in group 2 animals. One also needs to consider the role of pre-infection determinants, including previous infections, non-Coronavirus-related vaccinations and host genetic factors. Altogether our study demonstrates that SARS-CoV-2 infection can be associated with the induction of adaptive NK cells in lung-associated tissue.

The adaptive NK cells in the lung of the convalescent group 2 monkeys had a more differentiated, cytotoxic pro le (NKG2A^low^CD49a^+^CD69^+^GzB^+^CD107a^+^), in line with other studies on adaptive NK cells^18,26^. Of note, the adaptive lung NK cells here also shared several phenotypic features with the subset of tissue-resident NK cells (trNK)^84,85^, including expression of CD49a and CD69. The human lung has been reported to be a site of adaptive NK cells expressing CD49a^24^. Also consistent with previous reports in humans, these adaptive lung NK cells differed from classical human trNK cells by low expression of CD103^24^. Altogether, we have identified here the induction and long-term persistence after SARS-CoV-2 infection of Spike-specific, adaptive NK cells in the lung based not solely on phenotype, but also on tissue localization and function.

It is of note, that the MHC-E dependent adaptive activity was cross-reactive against MERS-CoV and SARS-CoV Spike-derived peptides, while the activity against the control peptides did not change. The Spike proteins from distinct Coronaviruses are only weakly homologous at the nucleotide or amino acid level, but they share helix structure and high hydrophobicity in common^86^. This is in agreement with structural analyses demonstrating a broad tolerance for hydrophobic and polar amino acids in the primary pockets of human (HLA-E) and simian (Mamu-E) MHC-E molecules^44^. Thus, one would anticipate that these adaptive NK cells are also reactive against SARS-CoV-2 variants other than the Wuhan strain, including against variants of concern.

In summary, our study revealed an unexpected high frequency of long-term persisting SARS-CoV-2 Spike antigen in macrophages of the lung. It unraveled that some animals were capable to escape MHC-E mediated inhibition of NK cells by SARS-derived peptides and become highly reactive against SARS-CoV-2 Spike LS peptides. This capacity was associated with the induction of regulatory macrophages and was specifically present in the lung and not detectable in blood. Moreover, the adaptive NK cell activity was cross-reactive against MERS-CoV and SARS-CoV spike-LS derived peptides. Given the high potential of cross-reactivity, it will be interesting to study in the future, if these NK cells contribute to protection in SARS-CoV-2 infection against variants of concern, and if they are inducible by immunotherapies and/or vaccinations.

## Materials And Methods

### Human Blood

Blood samples from healthy donors were obtained from the French blood bank (Etablissement Français du Sang) as part of an agreement with the Institut Pasteur (C CPSL UNT, number 15/EFS/023). The study was approved by the Ethics Review Committee (Comité de protection des personnes) of Île-de-France VII. Fifty milliliter blood samples were obtained from twelve donors. All blood samples were collected before 2019 and PBMC were isolated using Ficoll gradient and immediately frrozen.

#### Monkeys

Cynomolgus macaques (*M. fasicularis*), aged 37–40 months and originating from Mauritian AAALAC-certified breeding centres, were used in this study. All macaques were housed in IDMIT infrastructure facilities (CEA, Fontenay-aux-Roses), under BSL-2 and BSL-3 containment when necessary (animal facility authorization D92-032-02, Prefecture des Hauts de Seine, France) and in compliance with European Directive 2010/63/EU, the French regulations and the Standards for Human Care and Use of Laboratory Animals of the Office for Laboratory Animal Welfare (OLAW, assurance number A5826–01, United States). The protocols were approved by the institutional ethical committee ‘Comité d’Ethique en Expérimentation Animale du Commissariat à l’Energie Atomique et aux Energies Alternatives’ (CEtEA 44) under statement number A20–011. The study was authorized by the ‘Research, Innovation and Education Ministry’ under registration number APAFIS#24434–2020030216532863v1.

The animals were healthy and seronegative for SIV, type D retrovirus, and simian T-cell lymphotropic virus type 1 at the time of infection and were housed in single cages within level 3 biosafety facilities after infection. At the inclusion in the study the average weight of the monkeys was between 3 and 6 kg. All monkeys were young adults with an average age of 3–5 years at inclusion. Both males and females were used. Sample collection was performed in random order. The investigators were not blinded while the animal handlers were blinded to group allocation.

#### Viruses

For the *in vivo* studies, SARS-CoV-2 virus (hCoV-19/France/lDF0372/2020 strain) was isolated by the National Reference Center for Respiratory Viruses (Institut Pasteur) as previously described^31^. Virus stocks used *in vivo* were produced by two passages on mycoplasma-free Vero E6 cells in Dulbecco’s modified Eagle’s medium (DMEM) without FBS, supplemented with 1% penicillin (10,000 U ml^−1^) and streptomycin (10,000 μg ml^−1^) and 1 μg ml^−1^ TPCK-trypsin at 37 °C in a humidi ed CO_2_ incubator and titrated on Vero E6 cells.

#### Tissue collections and processing

Whole venous blood was collected in ethylenediaminetetraacetic acid (EDTA) tubes. Peripheral blood mononuclear cells (PBMCs) were isolated by Ficoll density-gradient centrifugation. Cells were either immediately stained for flow cytometry or cryopreserved in 90% foetal bovine serum (FBS) and 10% dimethyl sulfphoxide (DMSO) and stored in liquid nitrogen.

The BAL fluids (BALF) were collected by immediate gentle aspiration after each aliquot and pooled in a sterile heparinate lithium container. BALF samples were centrifuged at 350 g for 10 min. The cells were washed with PBS and then mononuclear cells (MC) were separated by standard density-gradient centrifugation. The percentage of BAL fluid recovered was approximately 75% of the instilled fluid. The alveolar cellularity ranged from 50000 to 200000 cells per ml of fluid recovered. No statistically significant difference in cellularity was noticed between baseline and post-infection broncho-alveolar lavage (data not shown). To rule out potential side-effects, we have verified that repeated bronchoalveolar lavages in uninfected animals do not affect the percentages of cells among BALMC. During the experiment, the majority of BALMC that were recovered were macrophages (more than 80%).

#### Sars-CoV-2 viral RNA quantification in nasopharyngeal and tracheal samples

Upper respiratory (nasopharyngeal and tracheal) specimens were collected with swabs (Universal transport medium, Copan; or Viral Transport Medium, CDC, DSR-052–01). Tracheal swabs were performed by insertion of the swab above the tip of the epiglottis into the upper trachea at approximately 1.5 cm of the epiglottis. All specimens were stored between 2 °C and 8 °C until analysis. Viral copy numbers were determined by quantitative RT-PCR with a plasmid standard concentration range containing an *rdrp* gene fragment including the RdRp-IP4 RT–PCR target sequence. The limit of detection was estimated to be 460 copies as described previously^30^. The protocol describing the procedure is also available on the WHO website (https://www.who.int/docs/default-source/coronaviruse/real-time-rt-pcr-assays-for-the-detection-of-sars-cov-2-institut-pasteur-paris.pdf?sfvrsn=3662fcb6_2).

### Sars-CoV-2 viral RNA quantification in BALF cells

Sars-CoV-2 viral RNA quantification in BALF cells we performed as follow: Cryopreserved BALF cells were quick-thawed, centrifuged, and washed in 2% BSA solution in D-PBS. Cells were blocked for 5 min in 2% BSA and then incubated at room temperature for 30 min with anti-NKG2a/c Ab (clone z199) to isolate simian NK cells by positive selection using magnetic beads (Miltenyi biotec). The negative fraction, consisting predominantly of macrophages, was then centrifuged and total RNA was extracted using RNeasy mini KIT (Qiagen). Primer and probes for the real-time quantitative reverse transcriptase and polymerase chain reaction (RT–qPCR) were the same as described above and available on the WHO website (https://www.who.int/docs/default-source/coronaviruse/real-time-rt-pcr-assays-for-the-detection-of-sars-cov-2-institut-pasteur-paris.pdf?sfvrsn=3662fcb6_2). Relative quantification of the viral genome was performed by one-step RT–qPCR using the RNA to ct 1 step kit (applied biosystem, 4392938). Thermal cycling was performed in a StepOnePlus Real-Time PCR System (Applied Biosystems) in MicroAmp Fast Optical 96-well reaction plates. 2 Δ Δ Ct was calculated based on the mean of Ct measured for viral RNA using the nCoV-IP4 primers and 18s RNA Cts obtained for each monkey.

### Polychromatic flow cytometry

PBMCs and BALF cells were stained as previously described^87^. The antibodies are listed in Supplementary method Table 1. The anti-NKG2A antibody used recognizes both NKG2A and NKG2C on simian cells^88^. Flow cytometry acquisitions were done on a LSRFortessa (BD Biosciences) or Symphony (BD Biosciences). Intracellular staining was performed using BD Cyto x/Cytoperm^™^. The data were further analyzed using FlowJo 10.4.2 software (FlowJo, LLC, Ashland, OR, USA). Multiparametric analyses were performed using FlowJo plugins Phenograph (version 3.0), UMAP (version 3.1). Phenograph was performed using a K mean of 50. UMAP was performed using a value for nearest neighbors of 25 and a minimal value of 0.25.

### Cell culture

K562 (human HLA class I–negative erythroleukemia) (ATCC® CCL-243) as well as HLA-E transduced K562 cells were maintained in RPMI 1640 (Life Technologies) supplemented with 10% heat-inactivated foetal calf serum (FCS), 2 mM, l-glutamine, 100 U/ml penicillin, and 100μg/ml streptomycin.

NK cells were maintained in RPMI 1640 with Glutamax (Life Technologies) supplemented with 10% heat-inactivated FCS, 2 mM, l-glutamine, 100 U/ml penicillin, 100μg/ml streptomycin and 100 IU/ml of IL-2 and 10 ng/mL of IL-15.

### Antibody against SARS-CoV-2 Spike protein

The human monoclonal antibody Cv2.3194, which targets the SARS-CoV-2 receptor binding domain, was cloned from a blood memory B cell of a COVID19 convalescent, produced as a recombinant IgG1 by transient transfection of Freestyle 293-F cells (Thermo Fisher Scienti c), and purified by affinity chromatography using Protein G Sepharose® 4 Fast Flow beads (Citivia)^89^.

#### Immunofluorescence staining

Macrophages were isolated from bronchoalveolar lavage using positive selection with or the anti-CD64 monoclonal antibody-PE conjugated (clone10.1 Bd bioscience, USA) reveals by anti-PE microbeads (Miltenyi Biotec (USA), the staining and the positive selection of the cells was done according to the supplier’s instructions. Immediately after positive selection, cells were cultured at the concentration of 5*10^5^cells/ml in a 24-well tissue culture plate (Nunc,Roskilde, Denmark) in RPMI 1640 supplemented with FBS and GM-CSF, 10 ng/ml; IL-3,10 ng/ml; M-CSF, 100 ng/ml; IL-4 during 16 hours (h). Cells were then fixed with PFA 4% and staining was performed. Membrane *permeabilization* was induced by *Triton* X-100 and were stained with mouse anti-CD163 monoclonal antibody (1: 200, ab-100909, Abcam, USA) and anti-SARS-CoV-2 Spike protein (see below). After 2 hours of incubation, the excess of unbound primary antibody was washed off with primary buffered saline solution of PBS. The primary antibodies were detected by secondary donkey anti-mouse polyclonal antibody with Alexa Fluor® 488 and Streptavidin coupled with Alexa 568 for 1 h at 37 °C (1 : 200, Thermo Fisher Scienti c, USA.). The sections were mounted using Vectashield with DAPI (4’6-diamidino-2-phenylindole, Vector Laboratories, USA). Immunofluorescence was analysed under an CELENA® X High Content Imaging System (LOGOS BIOSYSTEMS, south Korea)

### Peptides and HLA-E Stabilization assay

Synthetic peptides biotinylated or not were purchased from *Proimmune* (United Kingdom) and dissolved in DMSO at the concentration of 2mg/ml. The peptides used were VL9 (VMAPRTVLL), HSP60 (QMRPVSRVL), The sequence of the coronavirus peptide used in the study are described in the figure 5. K562 and K562-E*0101 cells were incubated with synthetic peptides (3–300μM) at 37°C for 15–20 hours in serum-free AIM-V medium (GIBCO BRL) at a concentration of 1–3 10^6^ cells/ml. Control cultures were kept at 37 °C for over 16 hours without peptides. Cells were then harvested, washed in PBS and cell-surface expression of HLA-E was determined by incubation with PE-conjugated anti-HLA-E antibody, washed twice with PBS and fixed in 100 μl of Cyto x (BD Biosciences). Cells were acquired using a LSR II (BD Biosciences), and FlowJo software (version 9.6.4, Tree Star, Ashland, OR) was used for all analyses. Results were expressed either directly as mean fluorescent intensity (MFI).

#### NK cell isolation

Humans NKG2a^+^ NK-cells were purified (>90%) from frozen PBMCs by positive selection with antibody-coated magnetic beads (Myltenyi Biotec). NHP NKG2a/c^+^ NK-cells were purified (>90%) from freshly isolated tissues (ie BALF and blood) by positive selection with antibody-coated magnetic beads (Myltenyi Biotec). NK cells (10^6^ cell/mL) were cultured in RPMI 1640 containing GlutaMAX, 10% FCS, penicillin (10 IU/mL) and streptomycin (10 μg/mL) in the presence of IL-2 (Miltenyi Biotec) at 100 IU/mL and IL-15 at 5ng/ml (Miltenyi Biotec) (Culture media). Cells were left unstimulated over night before putting them in contact with their target cells.

#### MHC-E dependent NK cell viral suppressor assays

NK cell activity was determined through expression of cell surface CD107a, as previously described. K562-E*0101 cells were incubated with 50 μM of a given peptide at 26°C for 15–20 h. NK cells were co-cultured with 2 × 10^4^ K562-E*0101 target cells pulsed or not with peptides at a NK cell/target cell ratio of 5:1. Target cells were in parallel cultured in the absence of NK cells. Anti-CD107a antibody was added at the start of the assay, and GolgiStop and GolgiPlug (both BD Biosciences) were added 1 hour after start of the stimulation. After 8 hours of culture, cell suspensions were stained for viability and cytotoxic activity was analyzed through measurement of CD107a expression by flow cytometry.

### Statistics

The GraphPad Prism 7 (GraphPad Software, San Diego, CA) was used to analyze data and to perform statistical analyses. Statistical test used are described in the legend for each figure, Values of *p* < 0.05 were considered significant. NK cell populations were analyzed by the uniform manifold approximation and projection method provided by the FlowJo plugin version 3.1. The heatmap was calculated as Raw *z* score of the indicated markers within the clusters using FLASKI^90^. Dendrograms present the clustering of samples (columns) and markers (row), which is based on hierarchical clustering with Euclidean distance metric and average linkage.

## Figures and Tables

**Figure 3:** Resident NK cells accumulate in lung of monkeys with low frequency of Spike^+^ macrophages **(a)** Graphs showing the percentage of NK cells among CD45^+^ cells from control, group 1 and group 2 convalescent monkeys, in the indicated tissues. **(b, c, d)** Histogram and Graph showing the mean uorescence intensity (MFI) and the percentage, respectively, of the indicated markers expressed in NKG2_a/c_^+^ NK cells in the given tissues. For generating histograms, cells from each monkey of the indicated group were concatenated. Each group was plot on the same histogram to facilitate the comparison for each marker between groups. In each graph, each dot, square or triangle represents an individual monkey, bars represent the median, and the interquartile range is shown. For graph and histograms, healthy monkeys are in gray (n=5), animals from the group 1 in pink (n=7), and animals from group 2 in blue (n=8). For each graph, *p* values are determined by non-parametric Kruskal Wallis test.

## Data Availability

The authors declare that all other data supporting the findings of this study are available within the article and its raw data les, or are available from the authors upon request.
